# α-Hederin regulates macrophage polarization to relieve sepsis-induced lung and liver injuries in mice

**DOI:** 10.1515/med-2023-0695

**Published:** 2023-05-25

**Authors:** Junan Zeng, Guangyu Zhao

**Affiliations:** Department of Neonatology, Northwest Women’s and Children’s Hospital, Xi’an, Shaanxi Province, 710061, P.R. China; Department of Pediatrics, Xi’an Central Hospital, Xi’an, Shaanxi Province, 710003, P.R. China

**Keywords:** α-Hederin, macrophage polarization, sepsis, lung and liver injury, NF-κB activation

## Abstract

Sepsis is one of the most fatal inflammatory diseases with multiple organ failure caused by pathological infection. α-Hederin, a monodesmosidic triterpenoid saponin, has many biological activities including anti-inflammation. This study aimed to investigate the effect of α-Hederin on lung and liver injuries in septic mice. Mice underwent cecal ligation and puncture-induced sepsis were intraperitoneally injected with 0.3 or 3 mg/kg α-Hederin. α-Hederin treatment dose-dependently attenuated the lung and liver injuries in septic mice. Correspondingly, α-Hederin significantly decreased malondialdehyde production, increased the levels of superoxide dismutase and glutathione in lung tissues, reduced serum alanine aminotransferase and aspartate aminotransferase activities, and suppressed the levels of TNF-α and IL-6 in both tissues and in the serum. Moreover, α-Hederin augmented CD206 level and inhibited the productions of CD86 and iNOS in lung and liver tissues of septic mice. Importantly, p-p65/p65 was suppressed, whereas IκB was elevated by α-Hederin. In conclusion, α-Hederin could improve the lung and liver injuries in mice with sepsis by regulating macrophage M1/M2 polarization and inhibiting the activation of NF-κB signaling pathway.

## 1 Introduction

Sepsis is a systemic inflammatory response syndrome that is caused by the dysfunction of host defense against infection, often leading to multiple organ failure [1,2]. Sepsis affects about 1 out of 3 patients in the intensive care unit. Worldwide, it is estimated that more than 30 million cases of sepsis occur every year, and approximately 6 million deaths because of this cause [3]. Any infected patients have the potential to develop sepsis, and its incidence reaches as high as 1–2% in all hospitalized patients [4]. Notably, the morbidity of sepsis is even higher in children [5]. Although increasing investigation on the mechanism of sepsis in recent decades, there are few effective treatments [6]; hence, it is extremely urgent to find new therapeutic drugs.

Macrophages are innate immune cells that are nearly distributed in all tissues of the body. They derive from blood-borne monocytes or reside as resident macrophages in peripheral and central tissues. Macrophages play a vital role in the host defense by controlling innate and acquired immunities [7]. Under the stimulation of various factors, including inflammatory responses, macrophages are recruited to the diseased tissues and become polarized [8]. The phenotypes of macrophage polarization are mainly divided into classically activated M1 and alternatively activated M2, and M1/M2 polarization is tightly regulated by many molecular mechanisms, such as NF-κB activation [8]. M1 macrophages can secrete a variety of pro-inflammatory cytokines, for example, tumor necrosis factor (TNF)-α, inducible nitric oxide synthase (iNOS), and interleukin (IL)-6, to induce tissue damage; hence, this phenotype of macrophages is also known as pro-inflammatory macrophages. Conversely, M2 macrophages are known as anti-inflammatory macrophages because they produce anti-inflammatory cytokines [9]. It has been reported that macrophages affect the occurrence and development of sepsis [10].

α-Hederin is a monosaccharide chain pentacyclic triterpenoid saponin that is isolated from the leaves of Hedera or *Nigella sativa* [11,12]. Previous studies have demonstrated that α-Hederin has many pharmacological activities, such as antitumor [11], antifungal [13], and antispasmodic effects [14]. Moreover, the anti-inflammatory activity of α-Hederin was found [15]. However, the role of α-Hederin in sepsis remains unknown. In our research, we evaluated the effect of α-Hederin in sepsis-induced lung and liver injuries and explored the underlying molecular mechanism.

## 2 Materials and methods

### 2.1 Animal study

Male C57BL/6 mice (4- to 5-week old) were obtained from Charles River (Beijing, China). They were housed in a temperature-controlled room with 12 h light/dark cycles. All animal protocols were approved by the Institutional Animal Care and Use Committee of Xi’an Central Hospital (Approval ID: 2021-013). After acclimatization for 3 days, all animals were randomly assigned to sham, cecal ligation and puncture (CLP), CLP + α-Hederin-low, and CLP + α-Hederin-high groups (*n* = 6 per group). Septic mouse models were established using the CLP method as previously described [16]. Briefly, a laparotomy was performed after anesthesia with 2% isoflurane. The cecum was exposed and ligated below the ileocecal valve using a 4–0 silk suture. The cecum was punctured once with a 22-G needle, extruding a small amount of fecal material. Then, the cecum was returned to the abdominal cavity, and the wound was closed with a 4–0 silk suture. Animals were subcutaneously injected with 1 mL of normal saline for fluid resuscitation. Mice in the sham group were performed the same surgical procedures but not ligation or puncture. Mice in the CLP + α-Hederin-low and CLP + α-Hederin-high groups were injected intraperitoneally with 0.3 or 3 mg/kg α-Hederin, respectively, 2 h before CLP operation. The dose of α-Hederin used was referred to a previous study [17]. In addition, the toxicity of α-Hederin alone in normal mice was examined by evaluating its effect on the functions of lung and liver.

### 2.2 Serum analysis and enzyme-linked immunosorbent assay (ELISA)

The blood sample was collected 24 h following CLP surgery under anesthetization with sodium pentobarbital (200 mg/kg). The blood was centrifuged at 1,500*g* for 10 min at 4°C to obtain serum. Serum levels of alanine aminotransferase (ALT) and aspartate aminotransferase (AST) were examined by commercial assay kits (Nanjing Jiancheng Bioengineering Institute, Nanjing, China). Serum levels of TNF-α and IL-6 were analyzed by ELISA kits specific for mice (R&D Systems, Minneapolis, MN, USA). Moreover, ELISA was also used to determine the levels of TNF-α and IL-6 in tissue homogenates of the lung and liver, which were homogenized in phosphate-buffered saline and centrifuged at 5,000*g* for 15 min at 4°C. In addition, levels of glutathione (GSH), malondialdehyde (MDA), and superoxide dismutase (SOD) in lung homogenates were assessed by commercially available assay kits obtained from Nanjing Jiancheng Bioengineering Institute (Nanjing, China).

### 2.3 Histological examination and immunohistochemistry

After blood sample collection, tissues of lung and liver were obtained, fixed with 10% formalin for 24 h, and embedded in paraffin. The 5-μm sections were cut and placed on glass slides. To perform hematoxylin and eosin (H&E) staining, these samples were deparaffinized with xylene and rehydrated in graded ethanol. Subsequently, the sections were stained with H&E and observed under a light microscope (Carl Zeiss, Jena, Germany). Five random fields were selected from one slide for scoring, and morphological changes were scored according to a previous study [18].

For immunohistochemistry analysis, after rehydration in graded ethanol, sections were performed antigen retrieval with the microwave. Then, the slides were incubated with primary antibodies, including anti-CD86 (Cell Signaling Technology, Danvers, MA, USA; Cat. No. 19589, 1:300), anti-CD206 (Cell Signaling Technology, Cat. No. 24595, 1:500), and anti-p-NFκB p65 (p-p65; Santa Cruz Biotechnology, Santa Cruz, CA, USA; Cat. No. sc-166748, 1:200), followed by incubation with secondary antibodies and diaminobenzidine. Lastly, the nuclei were counterstained with hematoxylin. Images were obtained under a light microscope, and the expression level was calculated by the *H* score system [19].

### 2.4 Terminal deoxynucleotide transferase dUTP nick end labeling (TUNEL) analysis

The presence of apoptotic cells in liver tissues was detected by TUNEL staining with an In Situ Cell Death Detection kit (Roche, Basel, Switzerland). Briefly, the tissues on slides were digested with 20 μg/mL proteinase K. After washing, the slices were incubated with the TUNEL reaction solution and diaminobenzidine substrate. Hematoxylin was added to stain the cell nucleus. Images were taken under a light microscope.

### 2.5 Western blot

The lung and liver tissues were homogenized in RIPA lysis buffer (Beyotime, Shanghai, China) supplemented with 1 mM phenylmethanesulfonylfluoride. After centrifuging at 12,000*g* for 15 min at 4°C, the supernatants were collected to determine protein concentration using a BCA™ protein assay kit (Pierce, Bonn, Germany). Total protein (30 μg/lane) was separated by the sodium dodecyl sulfate-polyacrylamide gel electrophoresis and then transferred onto polyvinylidene fluoride membranes (Millipore, Billerica, MA, USA). Subsequently, the membranes were blocked by 5% non-fat milk and incubated with primary antibodies, including CD86 (Cell Signaling Technology, Cat. No. 19589, 1:1,000), CD206 (Cell Signaling Technology, Cat. No. 24595, 1:1,000), iNOS (Cell Signaling Technology, Cat. No. 13120, 1:1,000), NFκB p65 (p65; Santa Cruz Biotechnology, Cat. No. sc-8008, 1:800), p-p65 (Santa Cruz Biotechnology, Cat. No. sc-166748, 1:800), IκB (Cell Signaling Technology, Santa Cruz Biotechnology, Cat. No. 9242, 1:1,000), and GAPDH (Cell Signaling Technology, Cat. No. 5174, 1:1,000) at 4°C overnight. After washing, the membranes were incubated in horseradish peroxidase-conjugated goat anti-rabbit or goat anti-mouse IgG secondary antibody (1:5,000, Beyotime) for 45 min at room temperature. Finally, the bands were visualized by an electrochemical luminescence kit (Millipore). The expression of protein was quantified by ImageJ software with normalization to GAPDH.

### 2.6 Statistical analysis

Data were expressed as the mean  ±  standard deviation and analyzed by GraphPad Prism 8 software (GraphPad Software, Inc.). One-way analysis of variance followed by Tukey’s multiple comparison test was used for comparisons. *P* value less than 0.05 was considered statistically significant.

## 3 Results

### 3.1 α-Hederin exhibits no toxicity to the lung and liver in normal mice

**Figure 1:**
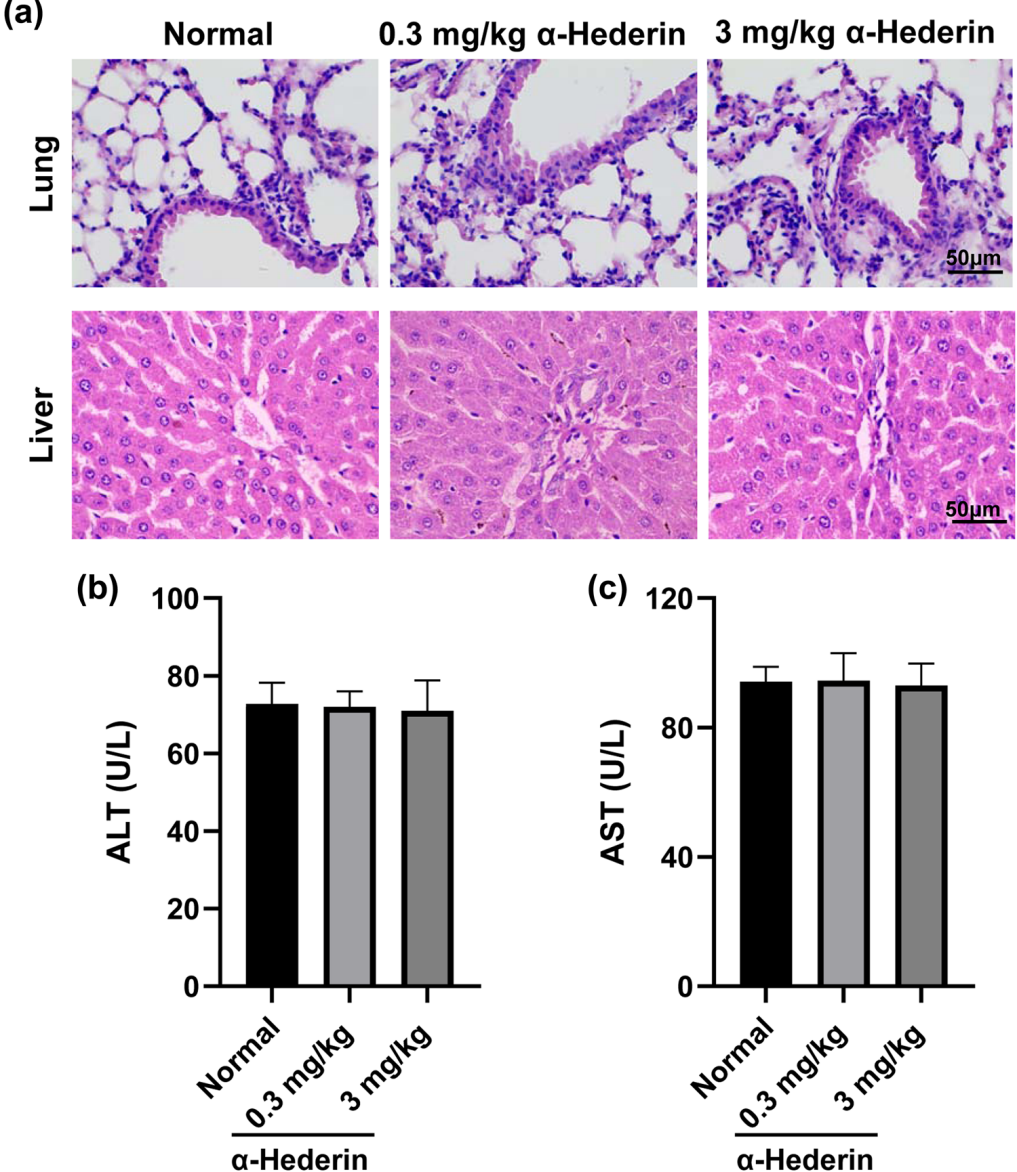
α-Hederin exhibits no toxicity to the lung and liver in normal mice. (a) H&E staining images of the lung and liver tissues from α-Hederin-treated normal mice. Scale bar = 50 μm. (b and c) Serum levels of ALT and AST in mice.

First, we evaluated the effect of α-Hederin on the lung and liver in normal mice. As indicated in Figure 1a, both mice treated with 0.3 and 3 mg/kg of α-Hederin showed normal structures in the lung and liver. Consistently, serum levels of ALT and AST were not altered by α-Hederin (Figure 1b and c). These results suggested that α-Hederin had no toxicity to the lung and liver in normal mice.

### 3.2 α-Hederin attenuates lung injury in mice with CLP-induced sepsis

**Figure 2:**
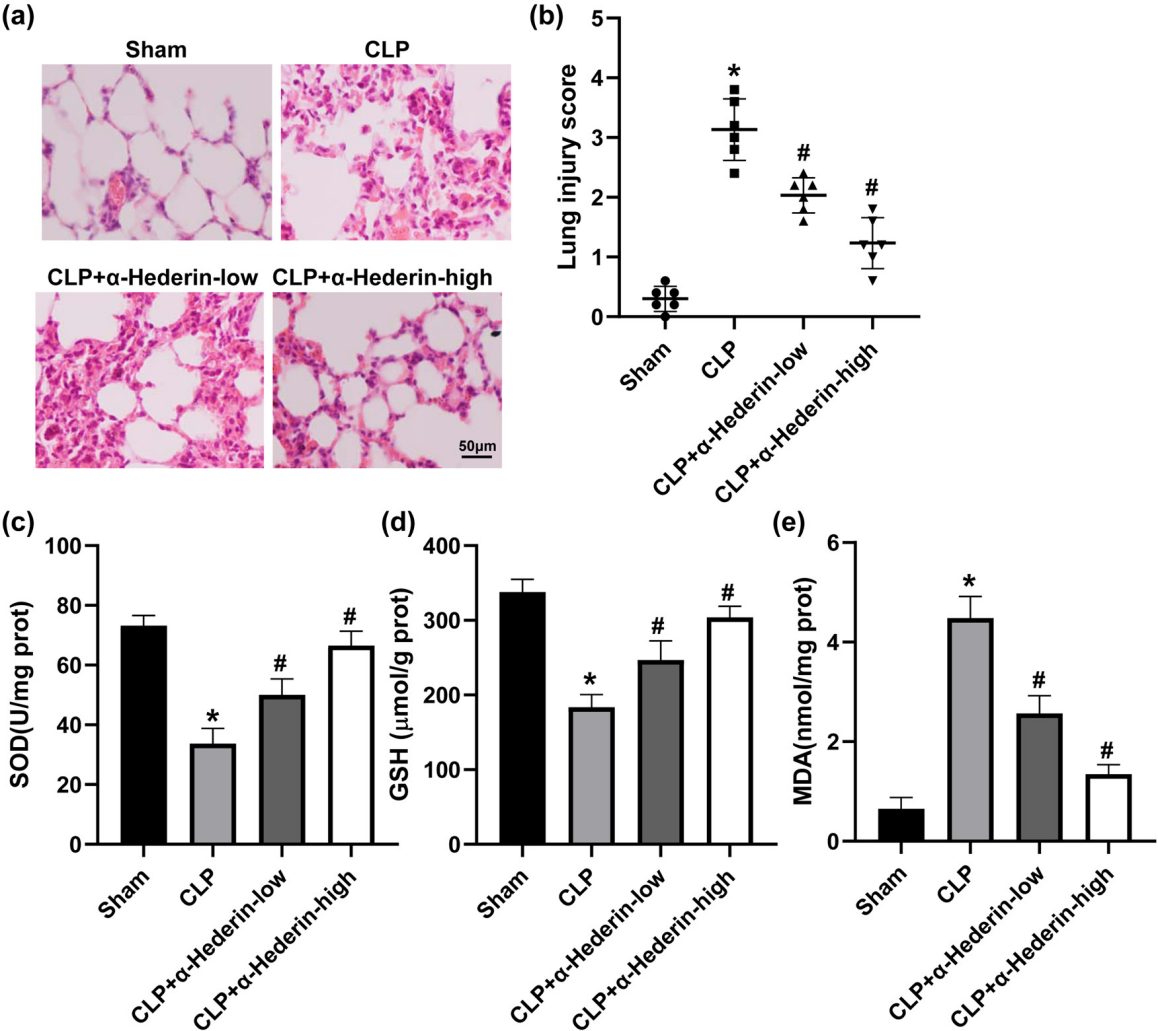
α-Hederin attenuates lung injury in CLP-induced septic mice. Mice were injected intraperitoneally with 0.3 or 3 mg/kg α-Hederin, respectively, 2 h before CLP operation. All mice were sacrificed 24 h after CLP surgery. (a) Representative histological analysis of lung of mice via H&E staining. Scale bar = 50 μm. (b) The injury of lung samples was calculated by semiquantitative analysis. The content of SOD (c), GSH (d), and MDA (e) in lung tissues. ^*^ *P* < 0.05 vs the sham group; ^#^ *P* < 0.05 vs the CLP group.

Histological analysis showed that a normal structure was exhibited in the lungs of the sham group. And CLP-induced mice revealed obvious inflammatory cell infiltration and interstitial thickness in the lung tissues. However, α-Hederin treatment dramatically decreased inflammatory cells and pulmonary interstitial thickening, with more efficiency at the high dose (Figure 2a). And the semiquantitative analysis confirmed that CLP significantly elevated the injury score, which was reduced by α-Hederin in a dose-dependent manner (Figure 2b). Furthermore, the levels of SOD and GSH in lung tissues suppressed by CLP were promoted by α-Hederin (Figure 2c and d). While α-Hederin significantly reduced the MDA production that was induced by CLP (Figure 2e). These data implied that α-Hederin attenuated lung injury in septic mice.

### 3.3 α-Hederin relieves liver injury in mice with CLP-induced sepsis

**Figure 3:**
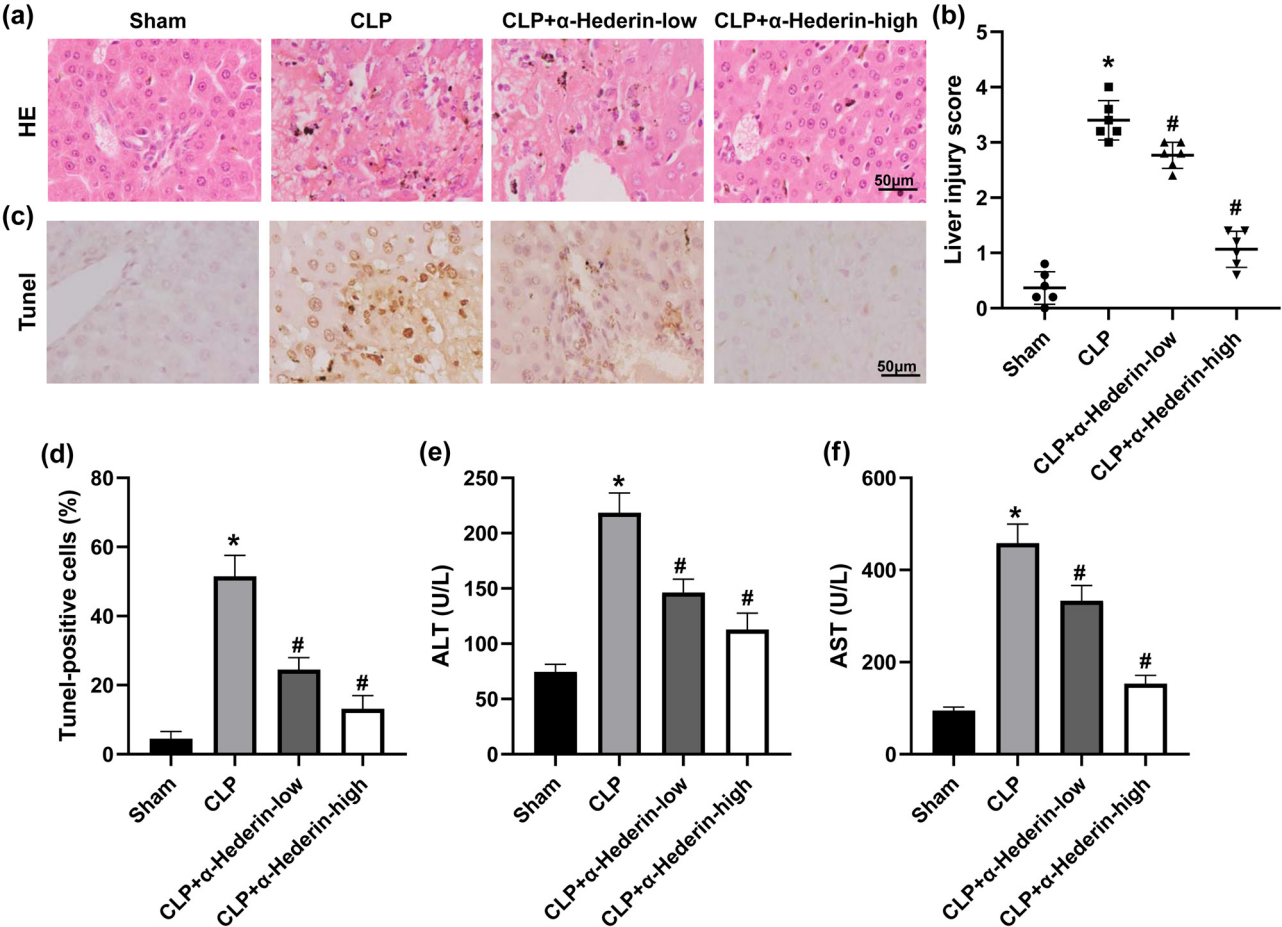
α-Hederin relieves liver injury in CLP-induced septic mice. (a) H&E staining images of liver tissues. Scale bar = 50 μm. (b) Semiquantitative analysis of the liver injury. (c and d) The apoptosis of hepatocytes was evaluated by TUNEL analysis and TUNEL-positive cells were calculated. Scale bar = 50 μm. ALT (e) and AST (f) levels were detected in the serum of mice under different treatments. ^*^ *P* < 0.05 vs the sham group; ^#^ *P* < 0.05 vs the CLP group.

The effect of α-Hederin on septic liver injury was also evaluated. Histological analysis revealed that CLP surgery significantly induced liver injury in mice, as indicated by swollen hepatocytes, obscure nucleus, vacuolar degeneration, and infiltration of inflammatory cells (Figure 3a and b). And α-Hederin treatment dramatically attenuated CLP-induced liver injury (Figure 3a and b). In line with this, the administration of α-Hederin notably reduced the apoptotic rate of hepatocytes induced by CLP (Figure 3c and d). Moreover, serum levels of ALT and AST, which are important liver function indexes, were prominently elevated by CLP, whereas inhibited by α-Hederin treatment (Figure 3e and f). Taken together, these results suggested that α-Hederin could attenuate the severity of liver injury in septic mice.

### 3.4 α-Hederin inhibits the inflammatory response in lung and liver tissues of septic mice

**Figure 4:**
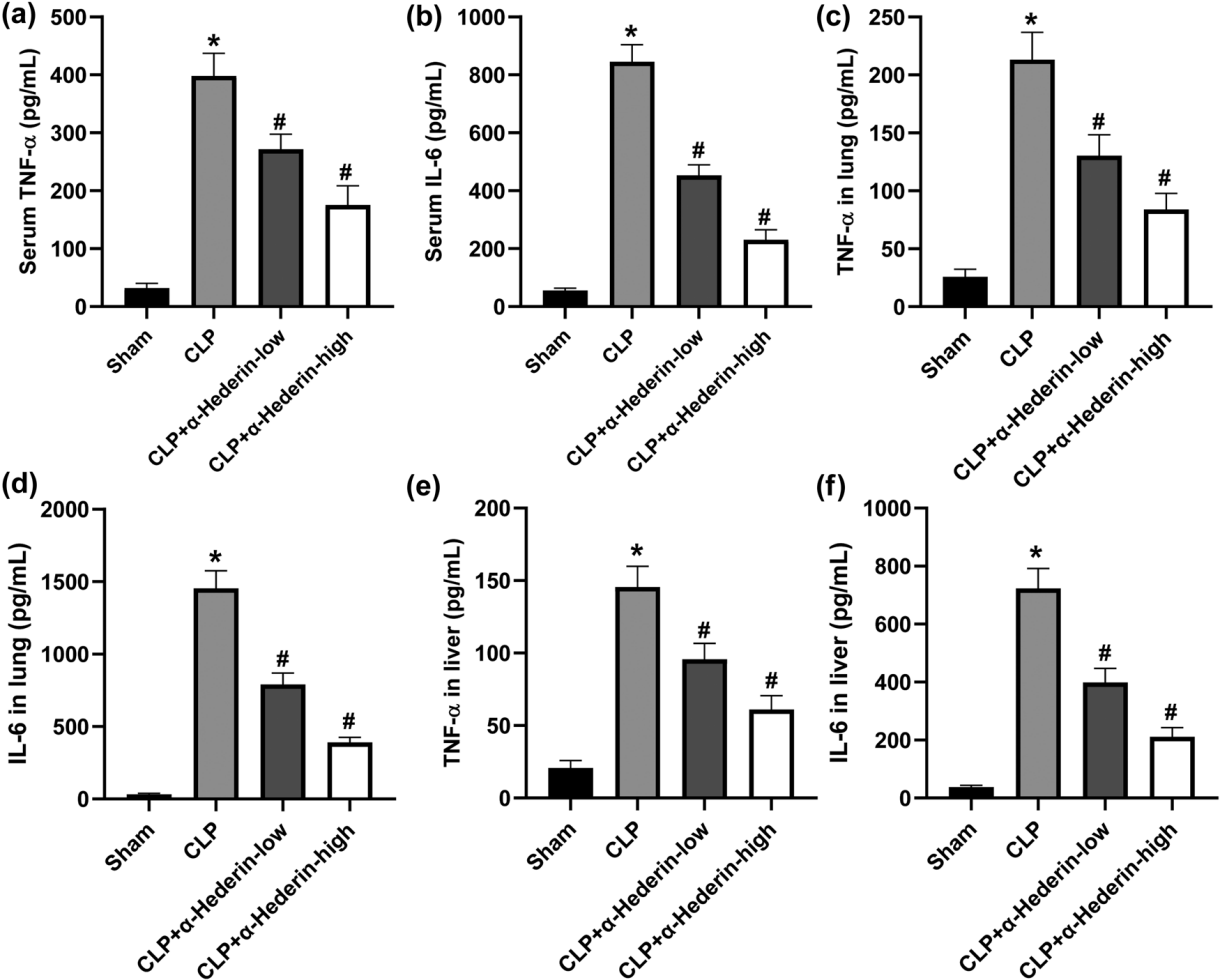
α-Hederin inhibits the inflammatory response in lung and liver tissues of septic mice. Serum levels of TNF-α (a) and IL-6 (b) in α-Hederin-treated septic mice. The levels of TNF-α (c) and IL-6 (d) in lung homogenates induced by CLP surgery were suppressed by α-Hederin. α-Hederin treatment significantly reduced the levels of TNF-α (e) and IL-6 (f) in the liver tissues of septic mice. ^*^ *P* < 0.05 vs the sham group; ^#^ *P* < 0.05 vs the CLP group.

To examine the potential effects of α-Hederin on inflammatory response in septic mice, serum levels of TNF-α and IL-6 were analyzed. As indicated in Figure 4a and b, CLP surgery-induced upregulation of TNF-α and IL-6 in serum were dose-dependently reduced by α-Hederin administration. Consistently, both the levels of TNF-α and IL-6 in lung and liver tissues were upregulated by CLP, the effects of which were inhibited by α-Hederin treatment (Figure 4c–f). These studies indicated that α-Hederin inhibited the inflammatory response in lung and liver tissues of septic mice.

### 3.5 α-Hederin regulates macrophage M1/M2 polarization

**Figure 5:**
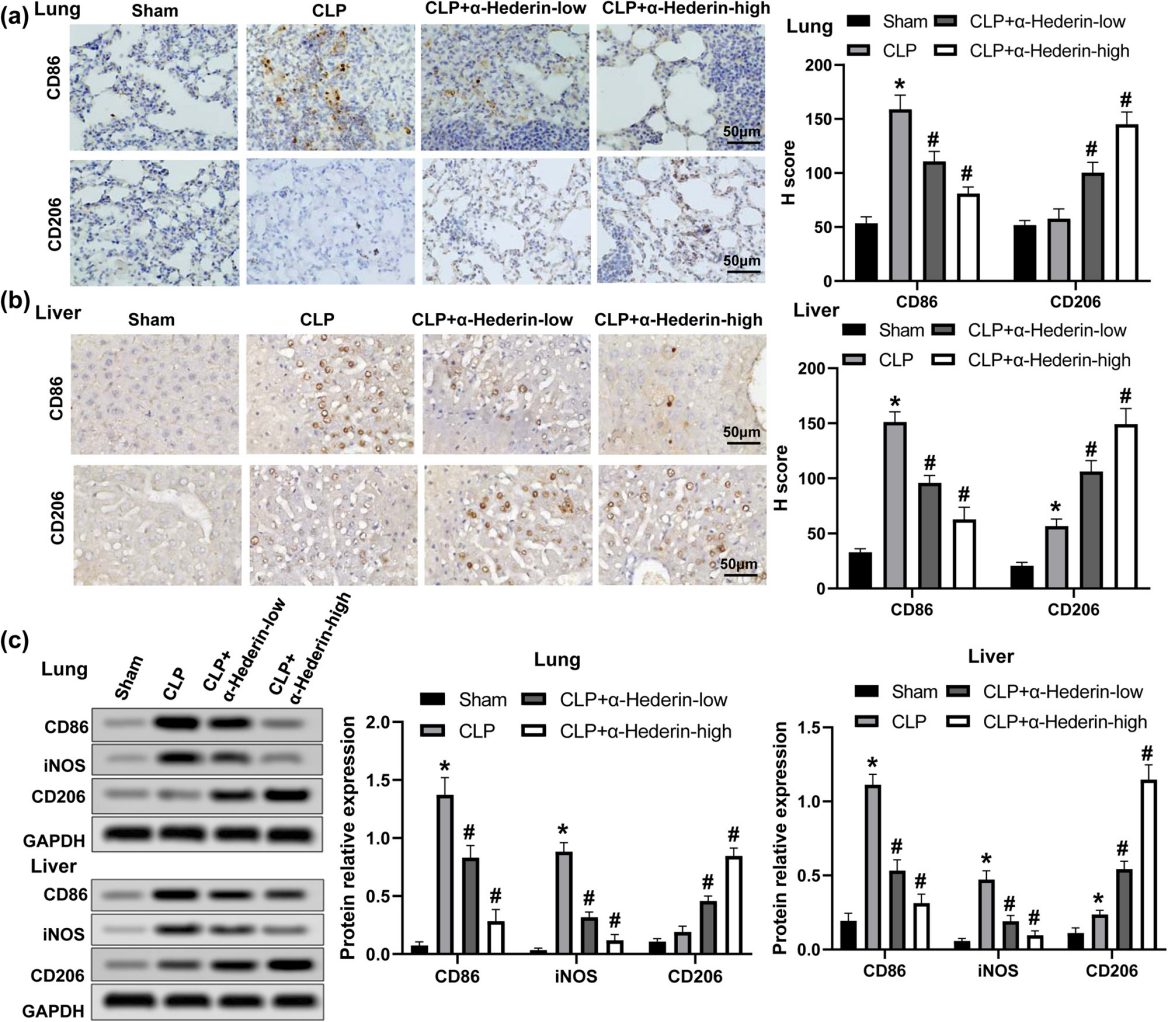
α-Hederin modulates the balance of M1/M2 macrophage polarization. Immunohistochemistry assay for the levels of CD86 and CD206 in lung (a) and liver (b) tissues. Scale bar = 50 μm. (c) Western blot was used to assay the protein levels of CD86, iNOS, and CD206 in the lung and liver tissues of mice. ^*^ *P* < 0.05 vs the sham group; ^#^ *P* < 0.05 vs the CLP group.

To investigate the role of α-Hederin on macrophage polarization phenotypes involving in sepsis-induced lung and liver injuries, levels of CD86 (M1 macrophage marker) and CD206 (M2 macrophage marker) under α-Hederin treatment were determined by immunohistochemistry assay. The results showed that CD86 was increased in CLP-induced mice both in the lung and liver tissues, and α-Hederin treatment decreased CD86 level induced by CLP (Figure 5a and b), whereas α-Hederin dramatically enhanced the level of CD206 in both tissues (Figure 5a and b). Next, the expression levels of M1 and M2 signature genes in lung and liver tissues were examined by western blot. As revealed in Figure 5c, compared with the CLP group, levels of iNOS and CD86 were suppressed, whereas CD206 protein level was increased in the α-Hederin-treated groups. These results suggested that α-Hederin could inhibit M1 macrophage polarization and promote M2 macrophage polarization in CLP-induced septic mice.

### 3.6 α-Hederin inhibits NF-κB activation in septic mice

**Figure 6:**
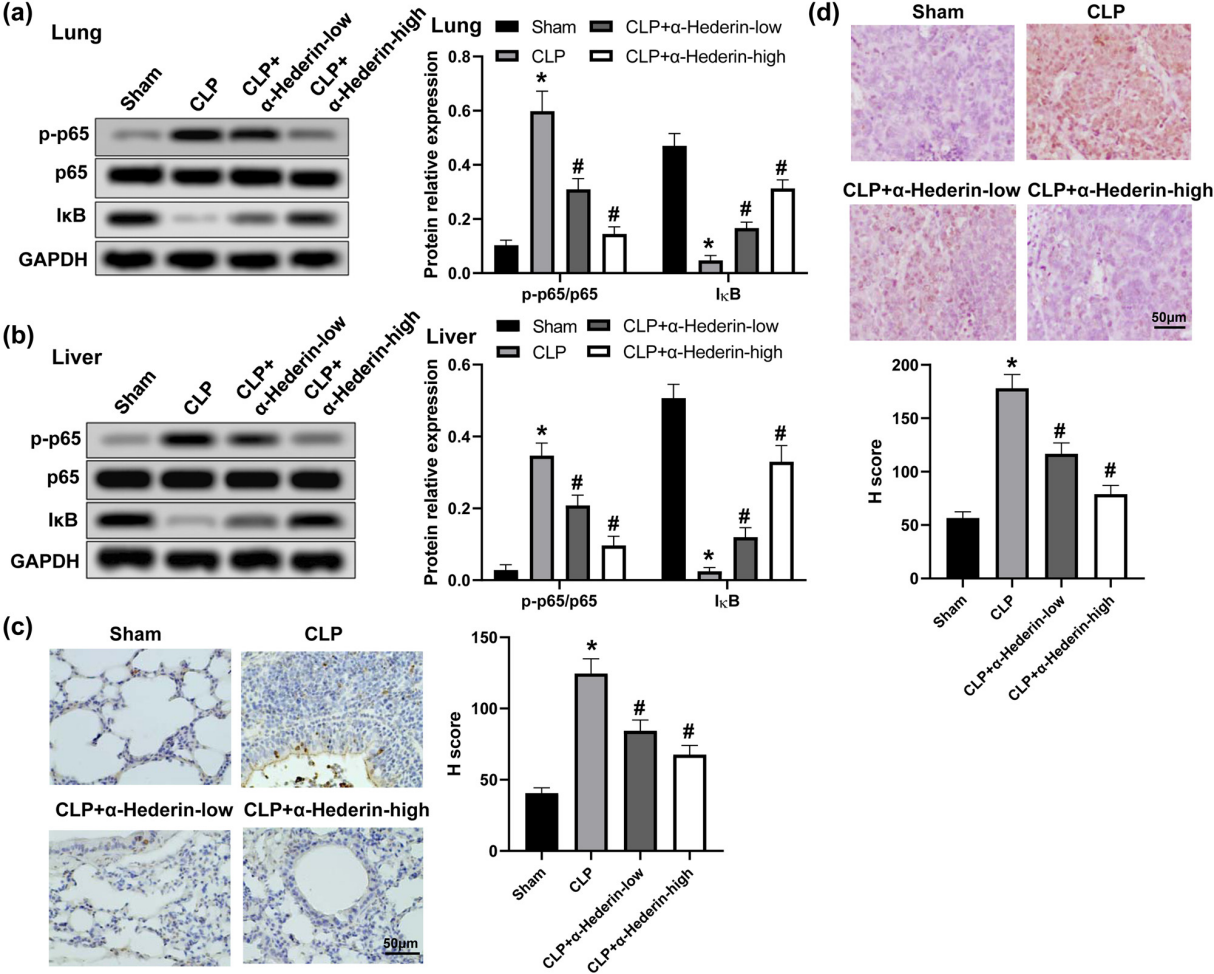
α-Hederin inhibits NF-κB activation in septic mice. The protein expression level of p-p65, p65, and IκB in the lung (a) and liver (b) tissues. p-p65 levels in the lung (c) and liver (d) samples were detected by immunohistochemistry, and their expression levels were calculated by the *H* score system. Scale bar = 50 μm. ^*^ *P* < 0.05 vs the sham group; ^#^ *P* < 0.05 vs the CLP group.

Studies have shown that NF-κB activation played an important role in the pathogenesis of organ injury induced by sepsis [20]. Hence, we investigated the effects of α-Hederin on the activation of NF-κB pathway. The results revealed that compared with the sham group, CLP notably increased the level of p-p65/p65 and reduced IκB expression both in lung and liver tissues, which was detected by western blot (Figure 6a and b). However, α-Hederin treatment decreased the level of p-p65/p65 and elevated IκB expression in a dose-dependent manner. In line with this result, immunohistochemistry analysis showed that α-Hederin administration inhibited p-p65 staining that was induced by CLP both in lung and liver tissues (Figure 6c and d). These results suggested that α-Hederin inhibited NF-κB activation in septic mice.

## 4 Discussion

Increasing evidence has revealed that natural products are promising candidate agents for sepsis because of their diverse biological activities and low toxicity to normal cells compared with antibiotics, which also cause antimicrobial resistance [21,22,23]. Our study found that α-Hederin had no toxicity to normal mice, whereas α-Hederin had strong inhibitory activity on the lung and liver injuries in CLP-induced septic mice. The inhibitory mechanism of α-Hederin was associated with the regulation of macrophage M1/M2 polarization and the inactivation of NF-κB signaling pathway.

The lung and liver are two vulnerable organs during sepsis [24,25]. Hence, we investigated the influence of α-Hederin on septic-induced lung and liver injuries. Previous studies demonstrated that α-Hederin inhibited lung inflammation in ovalbumin-sensitized rats and guinea pigs [15,17]. Jeong and Park [26] found that α-Hederin could protect mice from carbon tetrachloride-induced liver injury. Our results revealed that α-Hederin treatment significantly reduced CLP-induced the injuries of lung and liver, which was demonstrated by histological changes. Free radicals generated by oxidative stress play a crucial role in the development of multiple organ injuries [21,27]. MDA is a vital indicator of oxidative stress, whereas SOD and GSH are important markers of antioxidant stress [28]. Sepsis-induced multiple organ injuries are accompanied by an excessive response of innate inflammation, which stimulates the secretion of multiple inflammatory cytokines, such as TNF-α and IL-6 [27,29]. In this study, CLP significantly increased the MDA content and the secretion of TNF-α and IL-6, elevated the serum levels of ALT and AST, and decreased the activities of SOD and GSH. This confirmed that the sepsis model was successfully established. α-Hederin administration attenuated the MDA content and the secretion of TNF-α and IL-6 as well as serum levels of ALT and AST but elevated the activities of SOD and GSH that were changed by CLP operation. These findings suggested that α-Hederin attenuated the lung and liver injuries in septic mice.

Macrophages, important effector cells in innate immune response, play a critical role in inflammatory diseases. Upon stimulation, macrophages can differentiate into pro-inflammatory M1 phenotype and anti-inflammatory M2 phenotype [30]. Studies have shown that when an organ suffers from severe infection or inflammation, macrophages first polarized into M1 phenotypes to release large quantities of pro-inflammatory cytokines against the stimulus. However, if this status continues, M1 macrophages can cause tissue damage. Hence, M2 macrophages secrete anti-inflammatory factors to inhibit inflammation, which contributes to tissue repair and remodeling and retains homeostasis [9]. Disappointingly, in most instances, M2 macrophages are not enough to suppress the inflammation, leading to the initiation and development of many diseases [31]. Interfering with the activation status of macrophages is a promising therapeutic strategy in many diseases, including sepsis [32,33]. In this study, we found that α-Hederin significantly suppressed M1 macrophage polarization and promoted M2 macrophage polarization both in the lung and liver tissues of CLP-induced septic mice. This suggested that α-Hederin could restore the M1/M2 macrophage balance in sepsis.

NF-κB is one of the best-understood immune-related transcription factors, and abnormal activation of NF-κB plays a vital role in the pathogenesis of organ injury induced by sepsis [34,35]. In resting cells, it is inactive in the cytoplasm through interaction with inhibitory IκB proteins. Hyperactivation of NF-κB, phosphorylated NF-κB, is frequently observed after the phosphorylation of IκB proteins, which is stimulated by exogenous infection, endogenous damage, and tissue stress. Phosphorylated IκB is ubiquitinated and degraded, leading to the release of activated NF-κB to the nucleus [36]. Studies found that NF-κB regulated the development of sepsis by regulating macrophage polarization [37,38]. In the present study, we found that the level of p-p65 was decreased, while IκB expression was elevated by α-Hederin treatment in septic mice, demonstrating that α-Hederin suppressed the activation of NF-κB pathway in sepsis.

In conclusion, our study first demonstrated that α-Hederin exhibited the anti-inflammatory effect in sepsis-induced lung and liver injuries. α-Hederin administration dramatically suppressed M1 macrophage polarization and activated M2 macrophage polarization in CLP-induced septic mice, which was closely associated with the inactivation of NF-κB signaling pathway. However, the detailed molecular mechanisms and the potential clinical application of α-Hederin against sepsis are still needed to further investigate.
